# Methylation Patterns of *DKK1*, *DKK3* and *GSK3β* Are Accompanied with Different Expression Levels in Human Astrocytoma

**DOI:** 10.3390/cancers13112530

**Published:** 2021-05-21

**Authors:** Anja Kafka, Anja Bukovac, Emilija Brglez, Ana-Marija Jarmek, Karolina Poljak, Petar Brlek, Kamelija Žarković, Niko Njirić, Nives Pećina-Šlaus

**Affiliations:** 1 Laboratory of Neuro-Oncology, Croatian Institute for Brain Research, School of Medicine, University of Zagreb, Šalata 12, 10 000 Zagreb, Croatia; anja.bukovac@mef.hr (A.B.); brglez.e@gmail.com (E.B.); anamarija.jarmek@gmail.com (A.-M.J.); k.poljak96@gmail.com (K.P.); pbrlek@gmail.com (P.B.); njiricn@gmail.com (N.N.); nina@mef.hr (N.P.-Š.); 2 Department of Biology, School of Medicine, University of Zagreb, Šalata 3, 10 000 Zagreb, Croatia; 3 Department of Pathology, School of Medicine, University of Zagreb, Šalata 10, 10 000 Zagreb, Croatia; kamelijazarkovic@gmail.com; 4 Division of Pathology, University Hospital Center “Zagreb”, Kišpatićeva 12, 10 000 Zagreb, Croatia; 5 Department of Neurosurgery, University Hospital Center “Zagreb”, School of Medicine, University of Zagreb, Kišpatićeva 12, 10 000 Zagreb, Croatia

**Keywords:** astrocytic brain tumors, Wnt signaling, DKKs, GSK3β, APC, β-catenin

## Abstract

**Simple Summary:**

Astrocytomas are the most common type of primary brain tumor in adults. In this study, 64 astrocytoma samples of grades II–IV were analyzed for genetic and epigenetic changes as well as protein expression patterns in order to explore the roles of the Wnt pathway components, such as DKK1, DKK3, GSK3β, β-catenin, and APC in astrocytoma initiation and progression. Our findings on *DKK1* and *DKK3* show the importance of methylation in the regulation of Wnt signaling activity and also indicate pro-oncogenic effects of GSK3β on astrocytoma development and progression. Close connections between large deletions and mutations in the APC gene and increased β-catenin expression in glioblastoma were also established. Our results suggest that Wnt pathway related genes and proteins play an active role in the etiology of astrocytic brain tumors.

**Abstract:**

In the present study, we investigated genetic and epigenetic changes and protein expression levels of negative regulators of Wnt signaling, *DKK1*, *DKK3*, and *APC* as well as glycogen synthase kinase 3 (GSK3β) and β-catenin in 64 human astrocytomas of grades II–IV. Methylation-specific PCR revealed promoter methylation of *DKK1*, *DKK3*, and *GSK3β* in 38%, 43%, and 18% of samples, respectively. Grade IV comprised the lowest number of methylated *GSK3β* cases and highest of *DKK3*. Evaluation of the immunostaining using H-score was performed for β-catenin, both total and unphosphorylated (active) forms. Additionally, active (pY216) and inactive (pS9) forms of GSK3β protein were also analyzed. Spearman’s correlation confirmed the prevalence of β-catenin’s active form (r_s_ = 0.634, *p* < 0.001) in astrocytoma tumor cells. The Wilcoxon test revealed that astrocytoma with higher levels of the active pGSK3β-Y216 form had lower expression levels of its inactive form (*p* < 0.0001, Z = −5.332). Changes in *APC’s* exon 11 were observed in 44.44% of samples by PCR/RFLP. Astrocytomas with changes of *APC* had higher H-score values of total β-catenin compared to the group without genetic changes (t = −2.264, *p* = 0.038). Furthermore, a positive correlation between samples with methylated *DKK3* promoter and the expression of active pGSK3β-Y216 (r_s_ = 0.356, *p* = 0.011) was established. Our results emphasize the importance of methylation for the regulation of Wnt signaling. Large deletions of the *APC* gene associated with increased β-catenin levels, together with oncogenic effects of both β-catenin and GSK3β, are clearly involved in astrocytoma evolution. Our findings contribute to a better understanding of the etiology of gliomas. Further studies should elucidate the clinical and therapeutic relevance of the observed molecular alterations.

## 1. Introduction

Astrocytomas are glial cell tumors originating from astrocytes and account for nearly half of all primary brain tumors. According to the latest World Health Organization (WHO) classification, there are three different grades of astrocytoma, indicating their growth potential and aggressiveness [1,2]. Diffuse astrocytoma, defined as a grade II neoplasm, is a type of low-grade infiltrative glioma. Grade II astrocytomas have a tendency to progress toward high-grade malignancies called anaplastic astrocytomas (grade III) and eventually secondary glioblastomas (GBM, grade IV). The proliferative potential of diffuse astrocytomas and their growth rate are much lower than those of GBMs, which are a highly aggressive tumor with pronounced brain invasion and fast progression [1]. In addition to the biological behavior, an important criterion for the classification of diffuse glioma is the status of *IDH1* and *IDH2* gene mutations; astrocytomas are now defined as *IDH* mutant or *IDH* wild-type. Low-grade astrocytomas and secondary GBMs often carry *IDH* mutations, associated with younger age, as well as a much better prognosis [3,4]. *IDH* wild-type status refers to 90% of GBMs and indicates a primary tumor that arises de novo and carries a poorer prognosis than those classified as *IDH* mutant.

Despite new molecular findings that characterize tumors in the group of diffuse gliomas, the differences between individual pathohistological grades are still insufficiently investigated. For this reason, we decided to study the molecular characteristics of the WNT and AKT signaling pathway components in astrocytomas of different grades.

Signaling pathways form a complex network of molecular interaction in our cells, and a close connection between Wnt/β-catenin and PI3K/AKT/mTOR signaling has been described in many cancers [5]. One of the most prominent linking elements between these pathways is GSK3β (glycogen synthase kinase 3) [6]. The major mode of GSK3β activity regulation is through phosphorylation events. Activated Akt molecule phosphorylates GSK3β on the amino acid serine 9 (S9), leading to GSK3β inactivation. In contrast, GSK3β is activated by autophosphorylation or phosphorylation on tyrosine 216 (Y216) by other kinases [5,7]. In addition to S9 phosphorylation, promoter methylation may also be one of the mechanisms of GSK3β inactivation [8].

**Figure 1:**
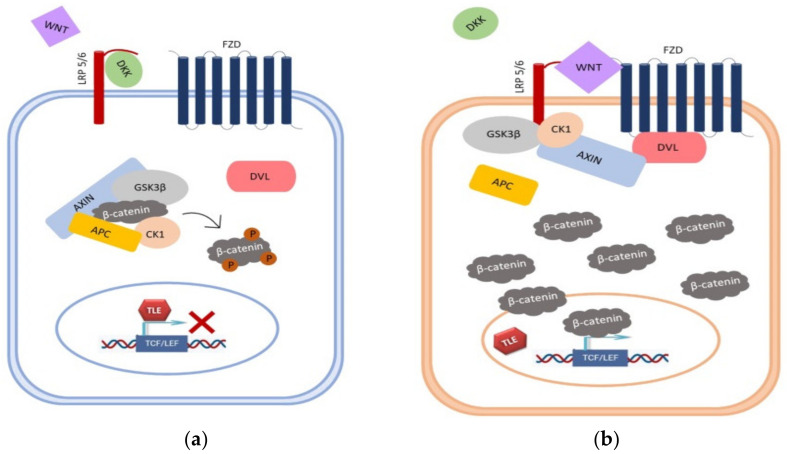
Overview of Wnt signaling pathway. (**a**) In the canonical Wnt pathway, DKK directly competes with Wnt for binding to LRP6. When DKK binds to the receptor, cytosolic pool of β-catenin is maintained at low levels through proteasomal degradation, due to its phosphorylation by the complex consisting of Axin/APC/CK1/GSK-3β. (**b**) Binding of Wnt to receptors Fz/LRP leads to the recruitment of components of the destruction complex to the membrane. This prevents phosphorylation and degradation of β-catenin, resulting in its accumulation in the cytoplasm. Stabilized β-catenin translocates into the nucleus and activates transcription of Wnt target genes.

In the Wnt signaling pathway, GSK3β plays a key role in modulating β-catenin and TCF/LEF (T cell factor/lymphoid enhancer-binding factor) transcription factor activity [7]. Active GSK3β can act as a tumor suppressor as it participates, together with other members of the destruction complex including APC (Adenomatous Polyposis Coli), AXIN1 and CK1 (Casein Kinase 1), in phosphorylation and subsequent degradation of the oncogenic β-catenin protein. In the pathway’s “off” state, TCF/LEF is inactive due to its interaction with the repressor Groucho. In contrast, inactive GSK3β stimulates cell proliferation in the pathway’s “on” state. The pathway is activated upon binding of Wnt ligands to the Frizzled (Fz) receptor and the co-receptor lipoprotein receptor-related protein (LRP) 5/6, resulting in the disintegration of the destruction complex and β-catenin cytoplasmic accumulation. Afterward, unphosphorylated β-catenin enters the cell nucleus, where it interacts with transcription factors from the TCF/LEF family, leading to Wnt target gene transcription (*cyclin D1*, *c-myc*, *fra-1*, *c-jun*, etc.) thus stimulating tumor growth [9]. Except for phosphorylating β-catenin, GSK3β can phosphorylate LRP co-receptor, thus revealing a binding site for AXIN on LRP, which mimics pathway activation by a Wnt ligand [10] (Figure 1).

Wnt signaling activity is also regulated by evolutionarily conserved inhibitors and activators that antagonize Wnt signaling such as the Dickkopf (DKK) gene family. The family consists of four members (DKK 1–4) in humans that specifically inhibit the Wnt/β-catenin signaling cascade by preventing the Wnt ligand from binding to LRP 5/6 co-receptors. Some members of the DKK family interact with transmembrane proteins Kremen 1 and 2, also modulating the pathway’s activity [11]. Not all DKK family members have consistent roles. Recent reports reveal that they can have dual agonistic and antagonistic functions, depending on the cellular context. Numerous studies report on changes in DKK protein expression within tumor tissues. DKK1 is differentially expressed in different types of human cancers, and its expression affects cell invasion, proliferation, and tumor growth. Some authors have reported on DKK1 overexpression [12,13,14,15,16,17,18,19,20,21], while others have noted its downregulation in tumors [22,23,24]. On the other hand, DKK3 is omnipresent in normal human tissues, including the brain; however, it is significantly depleted in various cancer cell types. DKK3 silencing due to epigenetic alterations has also been reported in multiple cancers [12]. However, there are few studies investigating the expression of DKK1 and DKK3 in gliomas [25,26,27,28].

These opposite reports indicate the need for further elucidation of the role of Wnt signaling molecules in cancer. Our study aims to contribute to the great efforts that are being made to clarify the genetic and epigenetic signatures in gliomas. Our goal was to clarify the behavior of *DKK1*, *DKK3*, and GSK3β and identify potential correlations to changes of *APC* and beta-catenin genes and proteins.

## 2. Materials and Methods

### 2.1. Tissue Samples

Sixty-four astrocytoma samples of different pathohistological types and grades, together with corresponding blood and formalin-fixed paraffin-embedded (FFPE) slides of brain tumor tissues, were collected with patients’ consents from the Departments of Neurosurgery and Departments of Pathology University Hospital Centers “Zagreb” and “Sisters of Charity”.

Chosen slides were reviewed by a certified pathologist (KŽ) to confirm the diagnosis (diffuse astrocytoma, anaplastic astrocytoma, glioblastoma). The diagnoses of astrocytic brain tumors were in concordance with the most recent WHO classification of the tumors of the central neural system [3]. In selected cases, additional immunohistochemical analyses (IDH1/2, ATRX, p53) were conducted in order to provide the correct diagnosis. The patients included in the study had no family history of brain tumors and did not undergo any cancer treatment prior to surgery, which could affect the results of molecular analyses. The sample consisted of 10 diffuse astrocytomas (grade II), 11 anaplastic astrocytomas (grade III), and 43 glioblastomas (grade IV). Twenty seven patients were female and 37 male. The age of patients varied from 6 to 83 (mean age = 50.31, median = 54 years). The mean age at diagnosis for females was 54.85 (median 56) and for males, 47 years (median 49).

The study was approved by the Ethical Committees, School of Medicine University of Zagreb (Case number: 380-59-10106-14-55/147; Class: 641-01/14-02/01) and University Hospital Centers “Sisters of Mercy” (number EP-7426/14-9) and “Zagreb” (number 02/21/JG, class: 8.1.-14/54-2). Patients gave their informed consent.

### 2.2. DNA Extraction

The genomic DNA extraction from unfixed frozen tumor tissue was performed according to the protocol by Green and Sambrook [29]. Briefly, approximately 0.5 g of tumor tissue was homogenized with 1 mL extraction buffer (10 mM Tris–HCl, pH 8.0; 0.1 M EDTA, pH 8.0; 0.5% sodium dodecyl sulfate) and incubated with proteinase K (100 µg/mL; Sigma-Aldrich, St. Louis, MO, USA) overnight at 37 °C. Organic (phenol–chloroform) extraction and ethanol precipitation followed.

The salting-out method was used to extract DNA from peripheral blood leucocytes [30]. Five milliliters of blood was lysed with 15 mL RCLB (red blood cell lysis buffer) (0.16 M NH4Cl; 10 mM KHCO3; 10 mM EDTA; pH 7.6), centrifuged (15 min/5000× *g*), and incubated overnight with 2 mL SE buffer (sodium-EDTA; 75 mM NaCl; 25 mM Na2EDTA; pH 8), 200 µL 10% SDS (sodium dodecyl sulphate) and 15 µL proteinase K (Sigma, Darmstadt, Germany) (20 mg/mL) at 37 °C. The salting-out method and isopropanol precipitation followed. The method is based on the principle that proteins and other cellular components, except DNA, will precipitate in a saturated salt solution (5M NaCl) due to their relative hydrophobicity.

The extracted DNA was successfully used for genetic (PCR/RFLP) and epigenetic (MS-PCR) analysis.

### 2.3. Polymerase Chain Reaction (PCR), Restriction Fragment Length Polymorphism (RFLP), Loss of Heterozygosity (LOH)

#### 2.3.1. Polymerase Chain Reaction

The PCR mixture (25 µL) for *APC*’s exon 11 amplification consisted of 10 pmol of each primer (5′-GGACTACAGGCCATTGCAGAA-3′ and 5′-GGCTACATCTCCAAAAGTCAA-3′), ~250 ng template DNA, 2.5 μL × 10x PicoMaxx reaction buffer, 2.5 mM of each dNTP, and 0.5 μL (1.25 U) of PicoMaxx high fidelity PCR system polymerase. PCR conditions were initial denaturation, 4 min/95 °C; denaturation, 1 min/94 °C; annealing, 2 min/58 °C; extension, 1.5 min/72 °C; for 35 cycles and final extension 7 min/72 °C. The PCR products were analyzed on 2% agarose gels.

#### 2.3.2. Restriction Fragment Length Polymorphism/Loss of Heterozygosity

Loss of heterozygosity of the *APC* gene was detected on the basis of restriction fragment length polymorphism (RFLP) of the PCR products. RFLP was performed by using restriction enzyme Rsa I, which recognizes a polymorphic site in exon 11 of the *APC* gene. PCR amplification of exon 11 generated a fragment of 133 bp that Rsa I cleaves into 85 bp and 48 bp fragments if the polymorphic site is present, or leaves uncleaved if the site is absent. LOH/Rsa I was demonstrated only in informative (heterozygous) samples when the tumor DNA showed loss of either the single uncut band (133 bp) or of the two cut bands (85 + 48 bp) compared to autologous blood tissue. PCR aliquots (20 µL) were digested with 6 U Rsa I (New England BioLabs, SAD) overnight at 37 °C and were electrophoresed on Spreadex EL 400 Mini gels (Elchrom Scientific, AL-Diagnostic GmbH, Amstetten, Austria) in the ORIGINS electrophoresis system (AL-Diagnostic GmbH, Amstetten, Austria) at 120 V and 55 °C.

#### 2.3.3. Methylation-Specific PCR (MSP)

After isolation from tumor tissue, DNA was treated with bisulfite using the MethylEdge Bisulfite Conversion System (Promega, Madison, WI, USA) following the manufacturer’s instructions. Bisulfite-treated DNA was afterward used for methylation-specific PCR (MSP).

Primer sequences of *DKK1*, *DKK3*, and *GSK3β* promoter region for MSP were synthesized according to [31,32,33], respectively (Table 1).

PCRs for bisulfite-treated DNA were performed using TaKaRa EpiTaq HS (TaKaRa Bio, USA): 1XEpiTaq PCR Buffer (Mg_2_^+^ free), 2.5 mM MgCl_2_, 0.3 mM dNTPs, 20 pmol of each primer (Sigma-Aldrich, USA), 50 ng of DNA, and 1.5 units of TaKaRa EpiTaq HS DNA Polymerase in a 25 µL final reaction volume. PCR cycling conditions are shown in Table 2.

PCR products were separated on 2% agarose gel stained with Syber Safe nucleic acid stain (Invitrogen, Thermo Scientific, Waltham, MA, USA) and visualized on a UV transilluminator. Methylated Human Control (Promega, Madison, WI, USA) was used as a positive control for the methylated reaction, while unmethylated DNA EpiTect Control DNA (Qiagen, Hilden, Germany) served as a positive control for the unmethylated reaction.

### 2.4. Immunohistochemistry (IHC)

Immunohistochemical staining was performed on 4 µm thick paraffin embedded tissue sections placed on silanized glass slides (DakoCytomation, Glostrup, Denmark). Tissue sections were deparaffinized in xylene (3×, 5 min), rehydrated in a decreasing ethanol series, (100%, 96%, and 70% ethanol; 2×, 3 min), and placed in water (30 s). Antigen retrieval was performed by heating the sections in microwave oven 2 times for 10 min at 400 W and 3 times for 5 min at 350 W in 6 M citrate buffer. Afterward, the endogenous peroxidase activity was blocked using 3% hydrogen peroxide for 10 min in dark. Non-specific binding was blocked by incubating samples with protein block serum-free ready-to-use (Agilent Technologies, Santa Clara, CA, USA) for 30 min at 4 °C. Next, sections were incubated with primary antibody Anti-GSK3β (phospho Y216) (rabbit polyclonal; ab75745, Abcam, Cambridge, MA, USA; dilution 1:100), Anti-GSK3β (phospho S9) (rabbit polyclonal; ab131097, Abcam, Cambridge, MA, USA; dilution 1:100), Active β-Catenin (rabbit monoclonal, non-phospho (Ser33/37/Thr41), D131A1, Cell Signalling Technology, Danvers, MA, USA; dilution 1:800) and Anti-Human Beta-Catenin (mouse monoclonal; Clone b-Catenin-1, M3539, Dako, Santa Clara, CA, USA; dilution 1:200) overnight at 4 °C. Dako REAL Envision detection system Peroxidase/DAB, Rabbit/Mouse, HRP (Agilent Technologies, Santa Clara, CA, USA) was used for visualization according to the manufacturer’s instructions, and the sections were counterstained with hematoxylin.

The level of expression of examined proteins in the healthy brain was determined by using the cerebral cortex of the human brain (Amsbio, Oxfordshire, UK). It was found that the level of immunoreactivity of all examined proteins in the healthy brain tissue was generally low, and the signal was detected only in the cytoplasm. Human placenta (decidual cells) and colon cancer served as positive controls. Negative controls underwent the same staining procedure but without incubating samples with primary antibodies.

### 2.5. Microscopic Analysis

In the tumor hot-spot area, 200 cells were counted and the intensity of protein expression was determined using the computer program ImageJ (National Institutes of Health, Bethesda, MD, USA). Astrocytic brain tumors stained for pGSK3β-Y216, pGSK3β-S9, β-catenin (non-phospho Ser33/37/Thr41), and total β-catenin protein were interpreted by 5 independent observers, of which 2 were pathologists using the following criteria: score 0 (no staining), score 1 (<10% tumor cells), score 2 (10–50% of tumor cells), and score 3 (>50% of tumor cells).

Next, a histological score (H-score) was calculated as the sum of the percentages of positively-stained tumor cells multiplied by the weighted intensity of staining:

H-score = [1 × (% of cells 1+) + 2 × (% of cells 2+) + 3 × (% of cells 3+)].

The H-score, therefore, ranged from 0–300, where ‘% of cells’ represents the percentage of stained cells for each intensity (1 = lack or weak expression, 2 = moderate expression, and 3 = strong expression).

Immunohistochemical results were interpreted blindly in regard to the genetic and epigenetic status.

### 2.6. Statistical Analysis

Statistical analysis was performed using SPSS v.19.0.1 (SPSS, Chicago, IL, USA) statistical program. The significance level was set at *p* < 0.05.

The distribution of the data was assessed by the Kolmogorov-Smirnov test and Shapiro-Wilk W-test. Depending on the results of the test of normality and the number of patients per group, differences in the values between the 3 grades were analyzed by one-way variance analysis (ANOVA) or Kruskal-Wallis test, while differences between the 2 groups were tested by Student’s t-test or the Mann-Whitney test. Pearson χ2 and Spearman’s correlation were used to test the relationships between *DKK1*, *DKK3*, and *GSK3β* methylation, *APC* genetic change, GSK3β and β-catenin protein expression levels, localization, and other clinical and demographic features.

## 3. Results

### 3.1. Methylation Status of Promoter Regions of GSK3β, DKK,1 and DKK3

**Figure 2:**
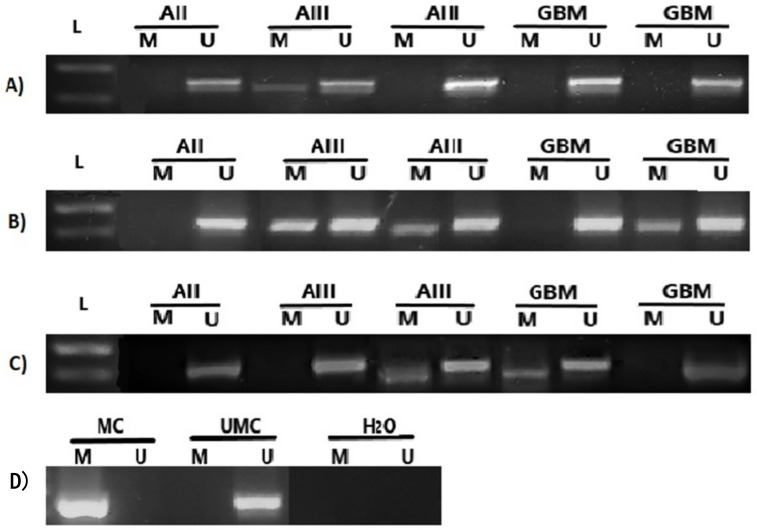
Methylation-specific PCR analysis for (**A**) *GSK3β*, (**B**) *DKK1*, and (**C**) *DKK3* promoter in astrocytic brain tumors grade II–IV. The presence of a visible PCR product in lanes marked M indicates the presence of methylated promoters, the presence of a product in lanes marked U indicates the presence of unmethylated promoters; (**D**) methylated human control (MC) was used as positive control for methylated reaction, unmethylated human control (UMC) was used as positive control for unmethylated reaction, and water served as negative control. L–standard DNA 50 bp ladder (Invitrogen).

Expression of *DKK1*, *DKK3*, and *GSK3β* genes is controlled, among other mechanisms, by DNA methylation (Figure 2).

**Figure 3:**
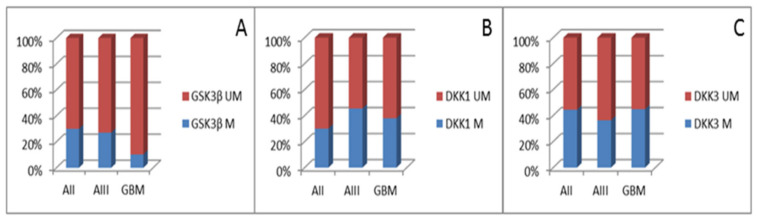
Graph showing the percentage of samples with methylated (M) and unmethylated (UM) promoter of (**A**) *GSK3β*, (**B**) *DKK1*, and (**C**) *DKK3* in astrocytic brain tumors grade II–IV.

Out of 50 analyzed astrocytoma samples of different grades, 41 (82%) had an unmethylated *GSK3β* gene promoter, while methylation of the promoter region was detected in nine samples (18%), including three AII (30%), three AIII (27%), and three GBM (10.34%) (Figure 3A). Furthermore, methylation of *DKK1* promoter was detected in 19 of 50 tumors (38%), including three AII (30%), five AIII (45.45%), and eleven GBM (37.93%), respectively. Similarly, *DKK3* promoter was methylated in 21 of 49 tumors (42.86%), including four AII (44.44%), four AIII (36.36%), and thirteen GBM (44.83%), respectively (Figure 3B,C). Although it was obvious that grade IV comprised the lowest number of methylated cases for *GSK3β* and highest for *DKK3*, the Kruskal-Wallis test showed no significant association of methylation patterns of *GSK3β* (*p* = 0.235)*, DKK1* (*p* = 0.771), and *DKK3* (*p* = 0.723) promoter regions with the tumor malignancy grade.

Spearman test did not reveal a statistically significant association of promoter methylation between *DKK1* and *DKK3* (*p* = 0.429), *DKK1* and *GSK3β* (*p* = 0.775), or *DKK3* and *GSK3β* (*p* = 0.121) in our sample (Figures S1–S4).

### 3.2. pGSK3β-S9 and pGSK3β-Y216 Expression Levels

**Figure 4:**
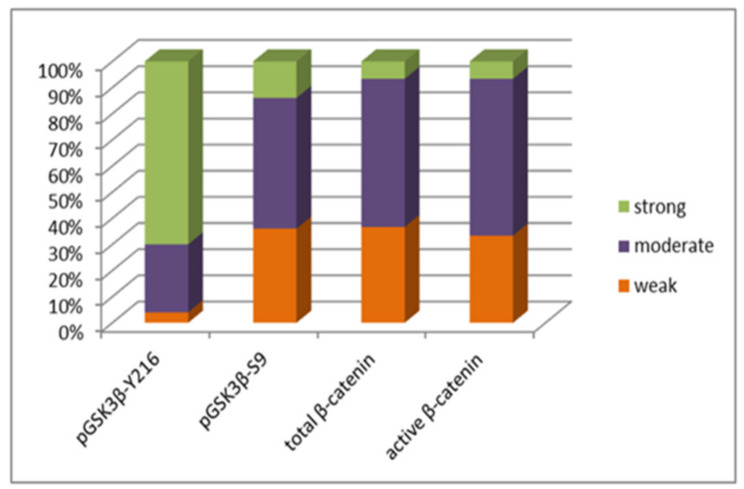
Graph illustrating the levels of expression of active (pGSK3β-Y216) and inactive (pGSK3β-S9) forms of GSK3β investigated in our total astrocytoma sample; and levels of expression of both forms of β-catenin (total and active) in glioblastoma group.

The effect of epigenetic changes on the protein expression levels was investigated in the same group of patients. When analyzing active pGSK3β-Y216 in the total sample, low expression was observed in 4% (2/50), moderate expression in 26% (13/50), and strong expression in 70% (35/50). Low expression of the inactive form, pGSK3β-S9, was present in 36% (18/50), moderate in 50% (25/50), and high in 14% (7/50) of total astrocytoma samples (Figure 4).

**Figure 5:**
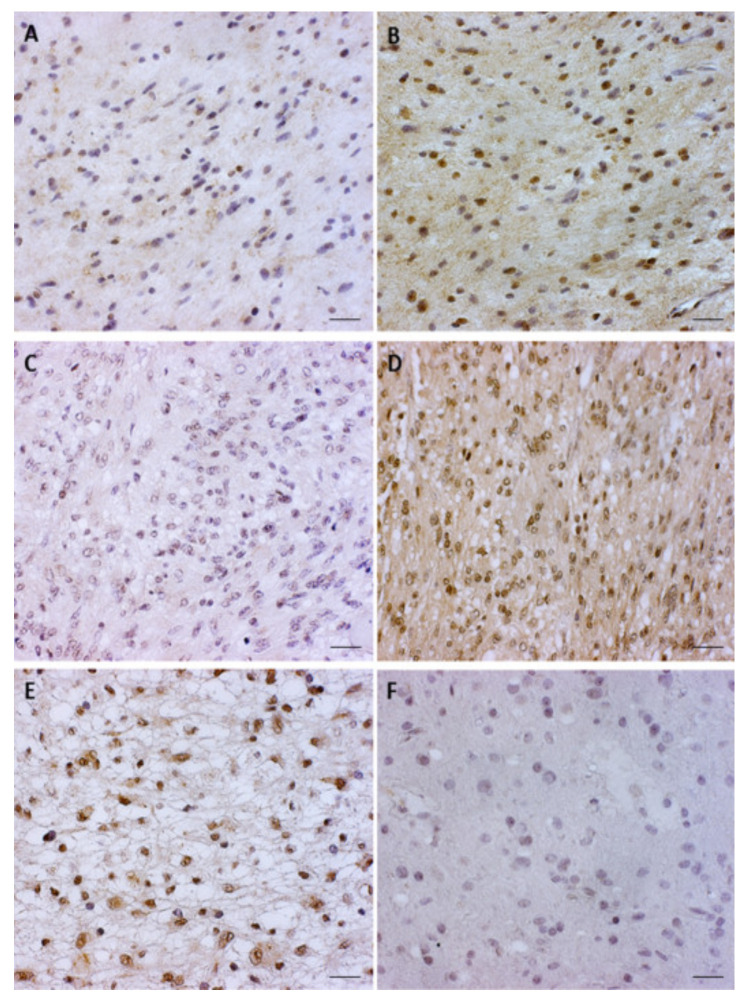
Characteristic immunohistochemical staining of active pGSK3β-Y216 and inactive pGSK3β-S9 protein in astrocytoma. (**A**) astrocytic brain tumor grade II with unmethylated GSK3β promoter showing weak cytoplasmic staining of pGSK3β-S9; (**B**) same astrocytic brain tumor grade II with unmethylated GSK3β promoter showing strong cytoplasmic and nuclear staining of pGSK3β-Y216; (**C**) glioblastoma (grade IV) with unmethylated GSK3β promoter showing lack of cytoplasmic staining of pGSK3β-S9; (**D**) same glioblastoma with unmethylated GSK3β promoter showing strong cytoplasmic and nuclear staining of pGSK3β-Y216; (**E**) glioblastoma (grade IV) with methylated GSK3β promoter showing moderate cytoplasmic and strong nuclear staining of pGSK3β-S9; (**F**) glioblastoma (grade IV) with methylated GSK3β promoter showing weak cytoplasmic staining of pGSK3β-Y216. Scale bar 50 µm.

When analyzing both forms of GSK3β expression in specific astrocytoma types, diffuse astrocytoma revealed 50% (5/10) of the samples with a lack or low levels of pGSK3β-S9 expression, while 70% (7/10) of the samples showed high levels of pGSK3β-Y216. In anaplastic astrocytoma, moderate expression was observed in 60% (6/10) of analyzed cases for pGSK3β-S9, while 70% (7/10) of samples showed a high level of pGSK3β-Y216 expression. The majority of glioblastoma samples analyzed for active pGSK3β-Y216 showed high levels of expression (70%), while for the inactive pGSK3β-S9, moderate expression was observed in 50% of samples. The signal was co-localized in the cytoplasm and cell nucleus in all of the analyzed samples (Figure 5).

Protein expressions of both active and inactive forms of GSK3β (pGSK3β-S9 (*p* = 0.728) and pGSK3β-Y216 (*p* = 0.820)) showed no significant association with any specific astrocytoma grade. Wilcoxon test revealed a statistically significant difference between the expression of pGSK3β-S9 and pGSK3β-Y216 protein in investigated astrocytoma (*p* < 0.0001, Z = −5.332). This result indicates that samples with a higher level of expression of active pGSK3β-Y216 have a lower expression level of inactive pGSK3β-S9 protein.

### 3.3. Total β-Catenin and Unphosphorylated β-Catenin Expression Levels in Glioblastoma

Expression and localization of total β-catenin (detects both phosphorylated and unphosphorylated form) and active (unphosphorylated) β-catenin were further examined in glioblastoma.

H-score analysis for total β-catenin revealed 36.66% (11/30) samples with weak, 56.67% with moderate (17/30) and 6.67% (2/30) with strong protein expression when compared to levels of β-catenin in a healthy brain (Figure 4). Grouped together, elevated expressions (2+ and 3+) were detected in 63.34% of samples. Most samples showed cytoplasmic expression (86.67%), while co-localization of the signal in cytoplasm and nucleus was present in only 4 cases (13.33%).

Unphosphorylated β-catenin showed a similar distribution of signal strengths. H-score analysis detected 33.33% (10/30) of samples with weak, 60% (18/30) with moderate, and 6.67% (2/30) with strong expression (Figure 4). When compared to cellular levels of β-catenin in a healthy brain, grouped elevated expressions (2+ and 3+) were observed in 66.67% of samples. Cytoplasmic and nuclear co-localization of unphosphorylated β-catenin was noticed in five samples (16.67%), and again in most samples, the expression was present exclusively in the cytoplasm (83.33%).

Spearman’s correlation showed a statistically significant positive correlation between H-score values of total and unphosphorylated β-catenin (r_s_ = 0.634, *p* < 0.001), which confirms the presence of an active form of β-catenin in tumor cells.

### 3.4. APC Exon 11 Genetic Changes in Glioblastoma

**Figure 6:**
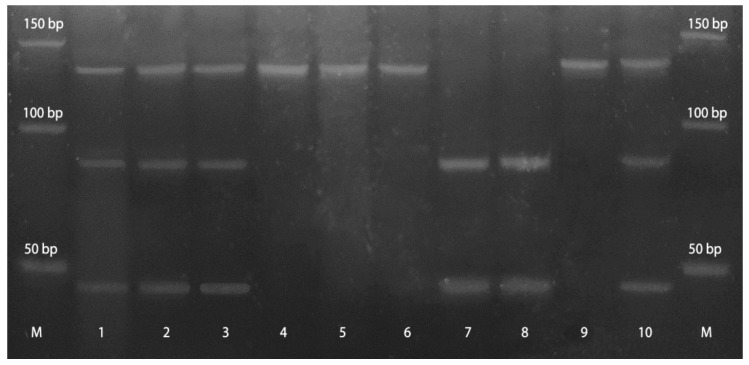
APC exon 11/RsaI/RFLP in glioblastoma samples is demonstrated. Lane M-standard DNA 50 bp ladder (Invitrogen); lanes 1, 2: heterozygous sample (tumor and blood), both alleles, cut and uncut, are visible; lane 3: possible restriction site introduced in tumor sample; lane 4: paired homozygous sample (blood); lanes 5, 6: homozygous sample (tumor and blood), uncut alleles are visible; lanes 7, 8: homozygous sample (tumor and blood), cut alleles are visible; lane 9: LOH, cut allele is missing; lane 10: corresponding informative blood sample, both alleles, cut and uncut, are visible.

Genetic changes of *APC* exon 11 were analyzed in 27 glioblastoma samples that were available for the analysis, nine (33%) of which were homozygous i.e., uninformative. Taking into account informative (heterozygous) samples, 44.44% (8/18) showed one of the two observed genetic changes. More precisely, LOH was detected in 33% (6/18) and the introduction of the restriction site because of mutation in 11.11% (2/18) cases (Figure 6).

Eight glioblastoma samples with genetic change in *APC* exon 11 showed moderate expression of total β-catenin in 62.5% and unphosphorylated β-catenin in 50% of samples. Student t-test revealed a significant difference of total β-catenin expression between groups with and without genetic changes in *APC’s* exon 11. Samples with changes had higher H-score values for total β-catenin, compared to the group without changes (t = −2.264, *p* = 0.038). However, a significant association of unphosphorylated (active) β-catenin H-score values with groups with or without *APC* genetic changes (U = 54.500, *p* = 0.197) could not be established.

### 3.5. The Correlations of Molecular Findings and Clinical Parameters

Spearman test revealed a significant positive correlation between *DKK3* methylation status and expression of active pGSK3β-Y216 (r_s_ = 0.356, *p* = 0.011), indicating that when the *DKK3* was epigenetically silenced, the expression of the active GSK3β was on the rise. Inactive pGSK3β-S9 protein was significantly positively correlated with the methylation of the GSK3β promoter region (r_s_ = 0.278, *p* = 0.050). Additionally, a bivariate correlation between active pGSK3β-Y216 and active (unphosphorylated) β-catenin showed a trend of negative association of the two proteins (r_s_ = −0.427, *p* = 0.088). Our analysis also showed that samples with genetic change in *APC* exon 11 were statistically significantly correlated with the increase of total β-catenin expression (r_s_ = 0.542, *p* = 0.004). The upregulation of β-catenin expression was noticed in 66% of analyzed samples. Of note is that 55% of the samples with upregulated β-catenin showed methylation in negative regulators of the signaling *DKK1* or *DKK3*.

Finally, no statistical significance was found between molecular findings and clinical parameters (*p* > 0.05), meaning that the analyzed molecular features were independent of the patients’ age and sex.

## 4. Discussion

The Wnt signaling pathway is frequently implicated in the etiology of various cancers and plays important roles in tumor initiation and progression. As recent reports indicate, impairment of negative regulators of Wnt signalization, i.e., DKKs, is often involved in tumor formation and growth [34]. Expression of the *DKK1* and *DKK3* gene is controlled, among other mechanisms, by DNA methylation, a common epigenetic silencing tool, which is increased in many tumors and tumor cell lines. Most research has indicated a DKK1 inhibitory effect in tumors [35,36,37,38], but interestingly, some studies showed DKK1′s tumor-promoting role [18,39]. Similarly, DKK3 was discovered to be downregulated in various types of malignant tissue [36,40,41,42,43,44], but there are also reports of DKK3 overexpression in hepatocellular and esophageal cancer [45,46,47].

Our findings on 50 astrocytoma samples revealed promoter methylation of *DKK1* in 38% and *DKK3* in 42.86% of the samples. Methylation of *DKK1* and *DKK3* was relatively constant across different grades (*DKK1*-AII 30%, AIII 45.45%, GBM 37.93%; *DKK3*-AII 44.44%, AIII 36.36%, GBM 44.83%). The remaining portion of astrocytoma samples did not show *DKK1* and *DKK3* promoter methylation; downregulation could be explained by the existence of additional epigenetic regulatory events. In the case of DKK1 and DKK3, these may be post-translational modifications in the histone tails, which are associated with transcriptional repression [35,48]. We also point out other mechanisms here that may contribute to *DKK1* and *DKK3* gene (and consequently DKK1 and DKK3 protein) silencing, such as the mutational burden and various miRNAs, as recently shown in a case of melanoma [49] and colorectal cancer [38,50,51].

A study by Götze et al. [27] suggests that primary and secondary GBMs are characterized by different *DKK1* and *DKK3* gene methylation profiles, helpful to distinguish between glioblastoma subtypes. In their study, promoter methylation of *DKK1* was quite rare in lower-grade astrocytoma but frequent in glioblastoma. Additionally, *DKK1* methylation was more frequently observed in secondary GBM (5/10), whereas none of the primary GBMs showed methylation of the *DKK1* promoter region. These findings suggest that methylation of *DKK1* may be linked to glioma progression and thus might be a potential prognostic marker. Furthermore, this study indicates that methylation of *DKK3* is a rare event in glioma, with no obvious association with the tumor type or grade [27]. Our research showed no significant association between methylation of *DKK1* (*p* = 0.767) or *DKK3* (*p* = 0.885) and the tumor grade or type. However, the frequency of methylation of the two genes was overall substantial, and it was shown that methylation of *DKK1* was higher in pooled grades III and IV (40%) in comparison to AII (30%). No significant association of promoter methylation between *DKK1* and *DKK3* (*p* = 0.429) was found in our study. Mizobuchi et al. [25] showed that DKK3 plays a pivotal role in regulating cell survival in human malignant glioma, promotes apoptosis, and facilitates the degradation of β-catenin. Similarly, there are also reports about a DKK1 pro-apoptotic function in glioma [36].

Apart from epigenetic silencing of negative regulators, aberrant pathway activity may be a result of the mutations in downstream components. Modifications that cause change in GSK3β activity, *CTNNB1* gene mutations targeting sites phosphorylated by GSK3β on β-catenin (S33, S37, and S41), and mutations of proteins that form a destruction complex with GSK3β (for example, APC) may all cause Wnt pathway hyperactivity [9].

The influence of GSK3β on tumor formation and promotion is still controversial. GSK3β can display both pro-oncogenic and tumor-suppressive effects as it has diverse roles in numerous cellular processes that also differ among different cell types [5].

In order to determine the character of the GSK3β role in astrocytoma grades II–IV, we examined the promoter methylation of the *GSK3β* gene and level of expression of active (pY216) and inactive (pS9) form of GSK3β protein. Although the proportion of methylated samples was relatively small in each astrocytoma grade, our results showed that methylation of *GSK3β* decreased with grade. In astrocytoma grade II, 30% of samples had methylated *GSK3β* promoter, followed by 27% of astrocytoma grade III and 10% of glioblastoma. Of note is that the number of unmethylated samples increased with grade, meaning that GSK3β is upregulated in aggressive cases. GBM is primarily diagnosed at older ages, and recent reports suggest that epigenetics, especially DNA methylation, seem to be age-dependent [52].

Immunohistochemical analysis revealed low expression levels of active pGSK3β-Y216 in 4% (2/50), moderate levels in 26% (13/50), and strong levels in 70% (35/50) of samples, which is consistent with the findings on unmethylated promoter. Expression of inactive pGSK3β-S9 was weak in 36% (18/50), moderate in 50% (25/50), and strong in 14% (7/50) of astrocytoma samples in grades II–IV. Wilcoxon test showed significant opposite levels of expression between pGSK3β-S9 and pGSK3β-Y216 protein in astrocytoma cells (*p* < 0.001, Z = −5.332), indicating that samples with a higher level of expression of active pGSK3β-Y216 have a lower expression level of inactive pGSK3β-S9 protein. Inactive pGSK3β-S9 protein was significantly positively correlated with methylation of *GSK3β* promoter (r_s_ = 0.278, *p* = 0.050), showing that in methylated cases, phosphorylation events also decrease protein expression. Similarly, Shakoori et al. [53,54] studied the expression of active and inactive forms of GSK3β in colorectal cancer. Although the study included a smaller number of samples, they proved that most patients had elevated expression of pGSK3β-Y216, whereas pGSK3β-S9 was mainly present in non-neoplastic tissues. Contrary to previously mentioned studies, high expression of inactive pGSK3β-S9 is found in skin [55], oral [56], and lung [57] cancers, which suggests tumor-suppressing effects of the enzyme in those malignant tissues.

The pro-oncogenic activity of GSK3β is based on the findings that deregulated GSK3β maintains tumor cell survival, proliferation, and invasion by enhancing machinery for cell motility and migration [58]. Finally, growing evidence marks GSK3β as a potential therapeutic target in cancer [59,60], thus encouraging the development of GSK3β inhibitors for cancer treatment [61]. In glioblastoma multiforme, such inhibitors facilitate apoptosis through inhibition of anti-apoptotic mechanisms in mitochondria and the NFkB pathway that is essential for cell survival [58,62].

Although GSK3β is generally considered a cytosolic protein, it can also be present in the nucleus. Our data showed an elevated level of expression of pGSK3β-Y216 in the cell nuclei of almost every sample of astrocytic brain tumors. It is known that GSK3β nuclear levels increase in response to apoptotic stimuli, and its major role is to affect gene expression by regulating the activity of many transcription factors [63].

The largest number of glioblastomas analyzed for both total and unphosphorylated β-catenin had moderate cytoplasmic expression (56.67% and 60%, respectively); weak expression was noted in 36.66% and 33.33%, respectively; while a strong signal was present in a smaller percentage of samples (6.67% and 6.67% respectively). Overall cytoplasmic accumulation of β-catenin predominated, whereas the expression of total and unphosphorylated β-catenin in the nucleus was observed in only four (13.33%) and five (16.67%) samples, respectively. It seems that strong expression and consequent transfer in the nucleus occurs in a smaller number of glioblastomas. Utsuki et al. [64] and Kahlert et al. [65] also found a small number of samples with nuclear expression, which can be partly explained by the Wnt pathway activity only in a small proportion of glioblastoma cells that have stem cell properties [66]. Another explanation is that the active form of beta-catenin that is transferred to the nucleus lacks specific epitopes and cannot be detected by this antibody. Spearman’s correlation showed a statistically significant positive correlation between H-score values of total and unphosphorylated β-catenin (r_s_ = 0.634, *p* < 0.001), thus confirming the presence of an active form of β-catenin in tumor cells. Phosphorylation status and localization of β-catenin are important indicators of the Wnt signaling pathway’s activation. Liu et al. [67] studied β-catenin expression in different grades of astrocytoma and noticed significantly higher levels of β-catenin in glioblastoma compared to lower grades and control groups, thus suggesting a role for β-catenin in the progression of malignant gliomas. The study by Sareddy et al. [68] on astrocytoma grade II–IV reports on the positive correlation of β-catenin mRNA and protein levels with the increase of malignancy grades. They also noticed a nuclear and cytoplasmic accumulation of β-catenin in astrocytoma, which is the hallmark of active Wnt/β-catenin signaling. Previous research by our laboratory has shown that tumors of neuroepithelial origin have higher levels of β-catenin expression compared to β-catenin expression levels in healthy brain tissue [69]. Kafka et al. [70] revealed that DVL3, TCF1, and LEF1 expression significantly increased with astrocytoma malignancy grades, suggesting their cooperation with nuclear β-catenin and joint involvement in malignant progression. In the present investigation, the bivariate correlation between unphosphorylated active β-catenin and active pGSK3β-Y216 showed a trend of negative association of the two proteins (r_s_ = −0.427, *p* = 0.088), confirming their mutually dependent relationship. Still, it seems that GSK3β activity toward β-catenin does not depend unambiguously on its phosphorylation status on S9, which appears to be a protective mechanism when GSK3β is aberrantly phosphorylated by some kinases [71,72]. It has been shown that highly active Akt does not fully inhibit GSK3β activity in some cancers and cancer cell lines [53]. However, a more recent study on human colorectal cancer cell lines shows that hyperactive Akt causes GSK3β inhibition and consequential β-catenin accumulation [73]. Recent studies demonstrated that active DKK3 is associated with reduced cytoplasmic and nuclear accumulation of β-catenin in different tumor types [74].

*APC* is a tumor-suppressor gene and an essential component of the beta-catenin complex that controls cytoplasmic beta-catenin levels. *APC* mutations occur early in gliomagenesis and result in increased beta-catenin levels that lead to the expression of Wnt responsive genes [9]. In our study, genetic change in *APC* exon 11 was present in 44.44% of the informative samples. Our laboratory group previously reported on *APC* exon 11 genetic changes in human brain tumors [75], brain metastases [76], and laryngeal squamous cell carcinoma [77] and found 33.3%, 58.8%, and 41% of samples with LOH or mutation of this gene, respectively. The present investigation also found that samples with changes of the *APC* gene had significantly higher values of total β-catenin H-score, compared to the group without genetic changes (t = −2.264, *p* = 0.038). Although the result for unphosphorylated β-catenin was not significant, its elevated expression in glioblastoma indicates the pathway’s activity and its association with genetic changes in *APC*. The relatively rare expression of β-catenin in the nucleus may also be explained by work from Morgan et al. [78], where they showed that APC loss alone was insufficient to stimulate nuclear β-catenin translocation, and further dysregulation is required. Another explanation for the rare β-catenin nuclear expression is the finding that most of the C-terminal deletions show the predominant nuclear localization [79], and the antibody that we used in our study was raised against the C-terminal β-catenin epitope.

In conclusion, the results of this study undoubtedly indicate the activation of the Wnt signaling pathway in astrocytoma. Our findings on *DKK1* and *DKK3* demonstrate the importance of methylation in the regulation of Wnt signaling activity but also suggest that additional regulatory mechanisms may be involved. Our findings indicate pro-oncogenic effects of GSK3β on astrocytoma development and progression not necessarily connected to the Wnt destruction complex. It is also evident that large deletions and mutations in the *APC* gene increase the level of β-catenin expression in glioblastomas. This research can provide more data about astrocytoma pathogenesis and help to better understand and improve the management of gliomas.

## Figures and Tables

**Table 1:** PCR primers used for MSP.

Primer		Sequence	Product Size
*DKK1*	MR-F	5′-CGTTCGTTGGTAGTTTTTATTTCGA-3′	175 bp
	MR-R	5′-GCGACTACCTTTATACCGCGAA-3′	
	UMR-F	5′-TGTTTGTTGGTAGTTTTTATTTTGA-3′	173 bp
	UMR-R	5′-ACCACAACTACCTTTATACCACAAA-3′	
*DKK3*	MR-F	5′-CGGTTTTTTTTCGTTTTCGGG-3′	154 bp
	MR-R	5′-CAAACCGCTACATCTCCGCT-3′	
	UMR-F	5′-TTTTGGTTTTTTTTTGTTTTTGGG-3′	155 bp
	UMR-R	5′-CCAA ACCACTACATCTCCACT-3′	
*GSK3β*	MR-F	5′ CGTCGTTATCGTTATCGTTC 3′	135 bp
	MR-R	5′ AATAACTCGAAAATACGACG 3′	
	UMR-F	5′ GAGGAGTTGTTGTTATTGTTATTGTTT 3′	136 bp
	UMR-R	5′ AAAAAAATAACTCAAAAATACAACA 3′	

MR-F and MR-R-primer set for methylated reaction; UMR-F and UMR-R-primer set for unmethylated reaction; bp-base pairs.

**Table 2:** MSP conditions for amplification of promoter region of *DKK1*, *DKK3*, and *GSK3β* genes.

Gene		Initial Denaturation	Cycle Conditions			Final Elongation
*DKK1*	MR	95 °C 5 min	95 °C 30 s	61 °C 30 s	72 °C 30 s	72 °C 7 min
	UMR	95 °C 5 min	95 °C 30 s	61 °C 30 s	72 °C 30 s	72 °C 7 min
*DKK3*	MR	95 °C 5 min	95 °C 30 s	61 °C 30 s	72 °C 30 s	72 °C 7 min
	UMR	95 °C 5 min	95 °C 30 s	61 °C 30 s	72 °C 30 s	72 °C 7 min
*GSK3β*	MR	95 °C 5 min	95 °C 30 s	55 °C 30 s	72 °C 30 s	72 °C 7 min
	UMR	95 °C 5 min	95 °C 30 s	56,5 °C 30 s	72 °C 30 s	72 °C 7 min

MR: methylated reaction; UMR: unmethylated reaction.

## Data Availability

Data supporting reported results are contained within the article. Some of the data presented in this study are available on request from the corresponding author. The data are not publicly available due to privacy issues.

## Supplementary Materials

The following are available online at https://www.mdpi.com/article/10.3390/cancers13112530/s1, Figures S1–S4: M = 50 bp molecular marker; MK-MR = methylated control methylated reaction; MK-UMR = methylated control unmethylated reaction; UMK-MR = unmethylated control methylated reaction; UMK-UMR = unmethylated control unmethylated reaction; S(1–6)-MR = sample (1–6) methylated reaction; S(1–6)-UMR = sample (1–6) unmethylated reaction; NK-MR = negative control methylated reaction; NK-UMR = negative control unmethylated reaction.

## References

[1] Perry A., Wesseling P. (2016). Histologic Classification of Gliomas. Handb. Clin. Neurol..

[2] Wesseling P., Capper D. (2018). WHO 2016 Classification of Gliomas. Neuropathol. Appl. Neurobiol..

[3] Louis D.N., Perry A., Reifenberger G., von Deimling A., Figarella-Branger D., Cavenee W.K., Ohgaki H., Wiestler O.D., Kleihues P., Ellison D.W. (2016). The 2016 World Health Organization Classification of Tumors of the Central Nervous System: A Summary. Acta Neuropathol..

[4] Kristensen B.W., Priesterbach-Ackley L.P., Petersen J.K., Wesseling P. (2019). Molecular Pathology of Tumors of the Central Nervous System. Ann. Oncol..

[5] Glibo M., Serman A., Karin-Kujundzic V., Bekavac Vlatkovic I., Miskovic B., Vranic S., Serman L. (2021). The Role of Glycogen Synthase Kinase 3 (GSK3) in Cancer with Emphasis on Ovarian Cancer Development and Progression: A Comprehensive Review. Bosn. J. Basic Med. Sci..

[6] Duda P., Akula S.M., Abrams S.L., Steelman L.S., Martelli A.M., Cocco L., Ratti S., Candido S., Libra M., Montalto G. (2020). Targeting GSK3 and Associated Signaling Pathways Involved in Cancer. Cells.

[7] Majewska E., Szeliga M. (2016). AKT/GSK3β Signaling in Glioblastoma. Neurochem. Res..

[8] Domoto T., Pyko I.V., Furuta T., Miyashita K., Uehara M., Shimasaki T., Nakada M., Minamoto T. (2016). Glycogen Synthase Kinase-3β is a Pivotal Mediator of Cancer Invasion and Resistance to Therapy. Cancer Sci..

[9] Bugter J.M., Fenderico N., Maurice M.M. (2021). Mutations and Mechanisms of WNT Pathway Tumour Suppressors in Cancer. Nat. Rev. Cancer.

[10] Zeng X., Tamai K., Doble B., Li S., Huang H., Habas R., Okamura H., Woodgett J., He X. (2005). A Dual-kinase Mechanism for Wnt Co-receptor Phosphorylation and Activation. Nature.

[11] Baetta R., Banfi C. (2019). Dkk (Dickkopf) Proteins. Arterioscler. Thromb. Vasc. Biol..

[12] Zhang K., Watanabe M., Kashiwakura Y., Li S.A., Edamura K., Huang P., Yamaguchi K., Nasu Y., Kobayashi Y., Sakaguchi M. (2010). Expression Pattern of REIC/Dkk-3 in Various Cell Types and the Implications of the Soluble Form in Prostatic Acinar Development. Int. J. Oncol..

[13] Shi R.Y., Yang X.R., Shen Q.J., Yang L.X., Xu Y., Qiu S.J., Sun Y.F., Zhang X., Wang Z., Zhu K. (2013). Expression of Dickkopfrelated Protein 1 is Related to Lymphatic Metastasis and Indicates Poor Prognosis in Intrahepatic Cholangiocarcinoma Patients after Surgery. Cancer.

[14] Begenik H., Kemik A.S., Emre H., Dulger A.C., Demirkiran D., Ebinc S., Kemik O. (2014). The Association between Serum Dickkopf-1 Levels and Esophageal Squamous Cell Carcinoma. Hum. Exp. Toxicol..

[15] Chen C., Zhou H., Zhang X., Ma X., Liu Z., Liu X. (2014). Elevated Levels of Dickkopf-1 are Associated with beta-catenin AccumuLation and Poor prognosis in Patients with Chondrosarcoma. PLoS ONE.

[16] Rachner T.D., Thiele S., Göbel A., Browne A., Fuessel S., Erdmann K., Wirth M.P., Fröhner M., Todenhöfer T., Muders M.H. (2014). High Serum Levels of Dickkopf-1 are Associated with a Poor Prognosis in Prostate Cancer Patients. BMC Cancer.

[17] Shi Y., Gong H.L., Zhou L., Tian J., Wang Y. (2014). Dickkopf-1 is a Novel Prognostic Biomarker for Laryngeal Squamous Cell Carcinoma. Acta Otolaryngol..

[18] Han S.X., Zhou X., Sui X., He C.C., Cai M.J., Ma J.L., Zhang Y.Y., Zhou C.Y., Ma C.X., Varela-Ramirez A. (2015). Serum Dickkopf-1 is a Novel Serological Biomarker for the Diagnosis and Prognosis of Pancreatic Cancer. Oncotarget.

[19] Sun D.K., Wang L., Wang J.M., Zhang P. (2015). Serum Dickkopf-1 Levels as a Clinical and Prognostic Factor in Patients with Bladder Cncer. Genet. Mol. Res..

[20] Shi X.D., Yu X.H., Wu W.R., Xu X.L., Wang J.Y., Xu L.B., Zhang R., Liu C. (2016). Dickkopf-1 Expression is Associated with Tumorigenity and Lymphatic Metastasis in Human Hilar Cholangiocarcinoma. Oncotarget.

[21] Watany M., Badawi R., Elkhalawany W., Abd-Elsalam S. (2017). Study of Dickkopf-1 (DKK-1) Gene Expression in Hepatocellular Carcinoma Patients. J. Clin. Diagn. Res..

[22] Jiang T., Wang S., Huang L., Zhang S. (2009). Clinical Significance of Serum DKK-1 in Patients with Gynecological Cancer. Int. J. Gynecol. Cancer.

[23] Liu Z., Sun B., Qi L., Li Y., Zhao X., Zhang D., Zhang Y. (2015). Dickkopf-1 Expression is Down-regulated during the Colorectal Adenoma-carcinoma Sequence and Correlates with Reduced Microvessel Density and VEGF Expression. Histopathology.

[24] Zhao Y.P., Wang W., Wang X.H., Xu Y., Wang Y., Dong Z.F., Zhang J.J. (2015). Downregulation of Serum DKK-1 Predicts Poor Prognosis in Patients with Papillary Thyroid Cancer. Genet. Mol. Res..

[25] Mizobuchi Y., Matsuzaki K., Kuwayama K., Kitazato K., Mure H., Kageji T., Nagahiro S. (2008). REIC/Dkk-3 Induces Cell Death in Human Malignant Glioma. Neuro Oncol..

[26] Zhou Y., Li W., Xu Q., Huang Y. (2010). Elevated Expression of Dickkopf-1 Increases the Sensitivity of Human Glioma Cell Line SHG44 to BCNU. J. Exp. Clin. Cancer Res..

[27] Götze S., Wolter M., Reifenberger G., Müller O., Sievers S. (2010). Frequent Promoter Hypermethylation of Wnt Pathway Inhibitor Genes in Malignant Astrocytic Gliomas. Cancer Genet..

[28] Oka T., Kurozumi K., Shimazu Y., Ichikawa T., Ishida J., Otani Y., Shimizu T., Tomita Y., Sakaguchi M., Watanabe M. (2016). A Super Gene Expression System Enhances the Anti-glioma Effects of Adenovirus-mediated REIC/Dkk-3 Gene Therapy. Sci. Rep..

[29] Green M.R., Sambrook J. (2012). Molecular Cloning—A Laboratory Manual.

[30] Miller S.A., Dykes D.D., Polesky H.F. (1988). A Simple Salting out Procedure for Extracting DNA from Human Nucleated Cells. Nucleic Acids Res..

[31] Maehata T., Taniguchi H., Yamamoto H., Nosho K., Adachi Y., Miyamoto N., Miyamoto C., Akutsu N., Yamaoka S., Itoh F. (2008). Transcriptional Silencing of Dickkopf Gene Family by CpG Island Hypermethylation in Human Gastrointestinal Cancer. World J. Gastroenterol..

[32] Zhang M., Huang M., Cao B., Sheng X., Li P. (2018). Methylation of the DKK3 Promoter is Associated with Poor Prognosis in Patients with Cervical Adenocarcinoma. Int. J. Clin. Exp. Pathol..

[33] Naghibalhossaini F., Zamani M., Mokarram P., Khalili I., Rasti M., Mostafavi-pour Z. (2012). Epigenetic and Genetic Analysis of WNT Signaling Pathway in Sporadic Colorectal Cancer Patients from Iran. Mol. Biol. Rep..

[34] Katoh M., Katoh M. (2017). Molecular Genetics and Targeted Therapy of WNT-related Human Diseases (Review). Int. J. Mol. Med..

[35] Aguilera O., Fraga M.F., Ballestar E., Paz M.F., Herranz M., Espada J., García J.M., Muñoz A., Esteller M., González-Sancho J.M. (2006). Epigenetic Inactivation of the Wnt Antagonist DICKKOPF-1 (DKK-1) Gene in Human Colorectal Cancer. Oncogene.

[36] Guo C.C., Zhang X.L., Yang B., Geng J., Peng B., Zheng J.H. (2014). Decreased Expression of dkk1 and dkk3 in Human Clear Cell Renal Cell Carcinoma. Mol. Med. Rep..

[37] Galamb O., Kalmar A., Peterfia B., Csabai I., Bodor A., Ribli D., Krenács T., Patai Á.V., Wichmann B., Barták B.K. (2016). Aberrant DNA Methylation of WNT Pathway Genes in the Development and Progression of CIMP-negative Colorectal Cancer. Epigenetics.

[38] Wang W., He Y., Rui J., Xu M.Q. (2019). miR-410 Acts as an Oncogene in Colorectal Cancer Cells by Targeting Dickkopf-related Protein 1 via the Wnt/β-catenin Signaling Pathway. Oncol. Lett..

[39] Shen Q., Fan J., Yang X.R., Tan Y., Zhao W., Xu Y., Wang N., Niu Y., Wu Z., Zhou J. (2012). Serum DKK1 as a Protein Biomarker for the Diagnosis of Hepatocellular Carcinoma: A Large-scale, Multicentre Study. Lancet Oncol..

[40] Urakami S., Shiina H., Enokida H., Kawakami T., Kawamoto K., Hirata H., Tanaka Y., Kikuno N., Nakagawa M., Igawa M. (2006). Combination Analysis of Hypermethylated Wnt-antagonist Family Genes as a Novel Epigenetic Biomarker Panel for Bladder Cancer Detection. Clin. Cancer Res..

[41] Yue W., Sun Q., Dacic S., Landreneau R.J., Siegfried J.M., Yu J., Zhang L. (2008). Downregulation of dkk3 Activates Beta-catenin/tcf-4 Signaling in Lung Cancer. Carcinogenesis.

[42] You A., Fokas E., Wang L.F., He H., Kleb B., Niederacher D., Engenhart-Cabillic R., An H.X. (2011). Expression of the Wnt Antagonist dkk3 is Frequently Suppressed in Sporadic Epithelial Ovarian Cancer. J. Cancer Res. Clin. Oncol..

[43] Park J.M., Kim M.K., Chi K.C., Kim J.H., Lee S.H., Lee E.J. (2015). Aberrant Loss of dickkopf-3 in Gastric Cancer: Can it Predict Lymph Node Metastasis Preoperatively?. World J. Surg..

[44] Lorsy E., Topuz A.S., Geisler C., Stahl S., Garczyk S., von Stillfried S., Hoss M., Gluz O., Hartmann A., Knüchel R. (2016). Loss of dickkopf 3 Promotes the Tumorigenesis of Basal Breast Cancer. PLoS ONE.

[45] Pei Y., Kano J., Iijima T., Morishita Y., Inadome Y., Noguchi M. (2009). Overexpression of Dickkopf 3 in Hepatoblastomas and Hepatocellular Carcinomas. Virchows Arch..

[46] Fujii M., Katase N., Lefeuvre M., Gunduz M., Buery R.R., Tamamura R., Tsujigiwa H., Nagatsuka H. (2011). Dickkopf (dkk)-3 and β-catenin Expressions Increased in the Transition from Normal Oral Mucosal to Oral Squamous Cell Carcinoma. J. Mol. Histol..

[47] Wang Z., Lin L., Thomas D.G., Nadal E., Chang A.C., Beer D.G., Lin J. (2015). The Role of Dickkopf-3 Overexpression in Esophageal Adenocarcinoma. J. Thorac. Cardiovasc. Surg..

[48] Valdora F., Banelli B., Stigliani S., Pfister S.M., Moretti S., Kool M., Remke M., Bai A.H.C., Brigati C., Hielscher T. (2013). Epigenetic Silencing of DKK3 in Medulloblastoma. Int. J. Mol. Sci..

[49] Huo J., Zhang Y., Li R., Wang Y., Wu J., Zhang D. (2016). Upregulated MicroRNA-25 Mediates the Migration of Melanoma Cells by Targeting DKK3 through the WNT/β-Catenin Pathway. Int. J. Mol. Sci..

[50] Rui Y., Hu M., Wang P., Zhang C., Xu H., Li Y., Zhang Y., Gu J., Wang Q. (2019). LncRNA HOTTIP Mediated DKK1 Downregulation Confers Metastasis and Invasion in Colorectal Cancer Cells. Histol. Histopathol..

[51] Guo J., Yang Z., Zhou H., Yue J., Mu T., Zhang Q., Bi X. (2020). Upregulation of DKK3 by miR-483-3p Plays an Important Role in the Chemoprevention of Colorectal Cancer Mediated by Black Raspberry Anthocyanins. Mol. Carcinog..

[52] Unnikrishnan A., Freeman W.M., Jackson J., Wren J.D., Porter H., Richardson A. (2019). The Role of DNA Methylation in Epigenetics of Aging. Pharmacol. Ther..

[53] Shakoori A., Ougolkov A., Yu Z.W., Zhang B., Modarressi M.H., Billadeau D.D., Mai M., Takahashi Y., Minalmoto T. (2005). Deregulated GSK3beta Activity in Colorectal Cancer: Its Association with Tumor Cell Survival and Proliferation. Biochem. Biophys. Res. Commun..

[54] Shakoori A., Mai W., Miyashita K., Yasumoto K., Takahashi Y., Ooi A. (2007). Inhibition of GSK-3 Beta Activity Attenuates Proliferation of Human Colon Cancer Cells in Rodents. Cancer Sci..

[55] Ma C., Wang J., Gao Y., Gao T.W., Chen G., Bower K.A., Odetallah M., Ding M., Ke Z., Luo J. (2007). The Role of Glycogen Synthase Kinase 3beta in the Transformation of Epidermal Cells. Cancer Res..

[56] Mishra R., Nagini S., Rana A. (2015). Expression and Inactivation of Glycogen Synthase Kinase 3 Alpha/Beta and their Association with the Expression of Cyclin D1 and p53 in Oral Squamous Cell Carcinoma Progression. Mol. Cancer.

[57] Zheng H., Saito H., Masuda S., Yang X., Takano Y. (2007). Phosphorylated GSK3beta-ser9 and EGFR are Good Prognostic Factors for Lung Carcinomas. Anticancer Res..

[58] Acikgoz E., Güler G., Camlar M., Oktem G., Aktug H. (2019). Glycogen Synthase Kinase-3 Inhibition in Glioblastoma Multiforme Cells Induces Apoptosis, Cell Cycle Arrest and Changing Biomolecular Structure. Spectrochim. Acta Part A Mol. Biomol. Spectrosc..

[59] McCubrey J.A., Steelman L.S., Bertrand F.E., Davis N.M., Sokolosky M., Abrams S.L., Montalto G., D’Assoro A.B., Libra M., Nicoletti F. (2014). GSK-3 as Potential Target for Therapeutic Intervention in Cancer. Oncotarget.

[60] Walz A., Ugolkov A., Chandra S., Kozikowski A., Carneiro B.A., O’Halloran T.V., Giles F.J., Billadeau D.D., Mazar A.P. (2017). Molecular Pathways: Revisiting Glycogen Synthase Kinase-3β as a Target for the Treatment of Cancer. Clin. Cancer Res..

[61] Sahin I., Eturi A., De Souza A., Pamarthy S., Tavora F., Giles F.J., Carneiro B.A. (2019). Glycogen Synthase Kinase-3β Inhibitors as Novel Cancer Treatments and Modulators of Antitumor Immune Responses. Cancer Biol. Ther..

[62] Kotliarova S., Pastorino S., Kovell L.C., Kotliarov Y., Song H., Zhang W., Bailey R., Maric D., Zenklusen J.C., Lee J. (2008). Glycogen Synthase Kinase-3 Inhibition Induces Glioma Cell Death through c-MYC, Nuclear Factor-kappaB, and Glucose Regulation. Cancer Res..

[63] Beurel E., Grieco S.F., Jope R.S. (2015). Glycogen Synthase Kinase-3 (GSK3): Regulation, Actions, and Diseases. Pharmacol. Ther..

[64] Utsuki S., Sato Y., Oka H., Tsuchiya B., Suzuki S., Fujii K. (2002). Relationship between the Expression of E-, N-cadherins and Beta-catenin and Tumor Grade in Astrocytomas. J. Neurooncol..

[65] Kahlert U.D., Suwala A.K., Koch K., Natsumeda M., Orr B.A., Hayashi M., Maciaczyk J., Eberhart C.G. (2015). Pharmacologic Wnt Inhibition Reduces Proliferation, Survival, and Clonogenicity of Glioblastoma Cells. J. Neuropathol. Exp. Neurol..

[66] Tompa M., Kalovits F., Nagy A., Kalman B. (2018). Contribution of the Wnt Pathway to Defining Biology of Glioblastoma. Neuromolecular Med..

[67] Liu X., Wang L., Zhao S., Ji X., Luo Y., Ling F. (2011). β-Catenin Overexpression in Malignant Glioma and Its Role in Proliferation and Apoptosis in Glioblastoma Cells. Med. Oncol..

[68] Sareddy G.R., Panigrahi M., Challa S., Mahadevan A., Babu P.P. (2009). Activation of Wnt/β-catenin/Tcf Signaling Pathway in Human Astrocytomas. Neurochem. Int..

[69] Nikuševa-Martić T., Pećina-Šlaus N., Kušec V., Kokotović T., Mušinović H., Tomas D., Zeljko M. (2010). Changes of AXIN-1 and Beta-catenin in Neuroepithelial Brain Tumors. Pathol. Oncol. Res..

[70] Kafka A., Bačić M., Tomas D., Žarković K., Bukovac A., Njirić N., Mrak G., Krsnik Ž., Pećina-Šlaus N. (2019). Different Behaviour of DVL1, DVL2, DVL3 in Astrocytoma Malignancy Grades and their Association to TCF1 and LEF1 Upregulation. J. Cell. Mol. Med..

[71] Woodgett J.R. (2001). Judging a Protein by more than Its Name: GSK-3. Sci. STKE.

[72] Ng S.S., Mahmoudi T., Danenberg E., Bejaoui I., de Lau W., Korswagen H.C., Schutte M., Clevers H. (2009). Phosphatidylinositol 3-kinase Signaling does not Activate the Wnt Cascade. J. Biol. Chem..

[73] Yun S.H., Park J.I. (2020). PGC-1α Regulates Cell Proliferation and Invasion via AKT/GSK-3β/β-catenin Pathway in Human Colorectal Cancer SW620 and SW480 Cells. Anticancer Res..

[74] Veeck J., Dahl E. (2012). Targeting the Wnt Pathway in Cancer: The Emerging Role of Dickkopf-3. Biochim. Biophys. Acta.

[75] Nikuševa-Martić T., Beroš V., Pećina-Šlaus N., Pećina H.I., Bulić-Jakuš F. (2007). Genetic Changes of CDH1, APC, and CTNNB1 Found in Human Brain Tumors. Pathol. Res. Pract..

[76] Pećina-Šlaus N., Martić T.N., Zeljko M., Bulat S. (2011). Brain Metastases Exhibit Gross Deletions of the APC Gene. Brain Tumor Pathol..

[77] Pećina-Šlaus N., Kljaić M., Nikuševa-Martić T. (2005). Loss of Heterozygosity of APC and CDH1 Genes in Laryngeal Squamous Cell Carcinoma. Pathol. Res. Pract..

[78] Morgan R.G., Ridsdale J., Tonks A., Darley R.L. (2014). Factors Affecting the Nuclear Localization of β-Catenin in Normal and Malignant Tissue. J. Cell. Biochem..

[79] Dar M.S., Singh P., Singh G., Jamwal G., Hussain S.S., Rana A., Akhter Y., Mong S.P., Dar M.J. (2016). Terminal Regions of β-catenin are Critical for Regulating Its Adhesion and Transcription Functions. Biochim. Biophys. Acta.

