# Supplementary material for: Methylation Patterns of DKK1, DKK3 and GSK3β Are Accompanied with Different Expression Levels in Human Astrocytoma
Source: Cancers (Basel). 2021 May 21;13(11):2530. doi: 10.3390/cancers13112530 (PMC8196684; doi:10.3390/cancers13112530)
Supplement: Supplementary file 1 [file cancers-13-02530-s001.zip › cancers-1227146-supplementary - Copy.pdf]

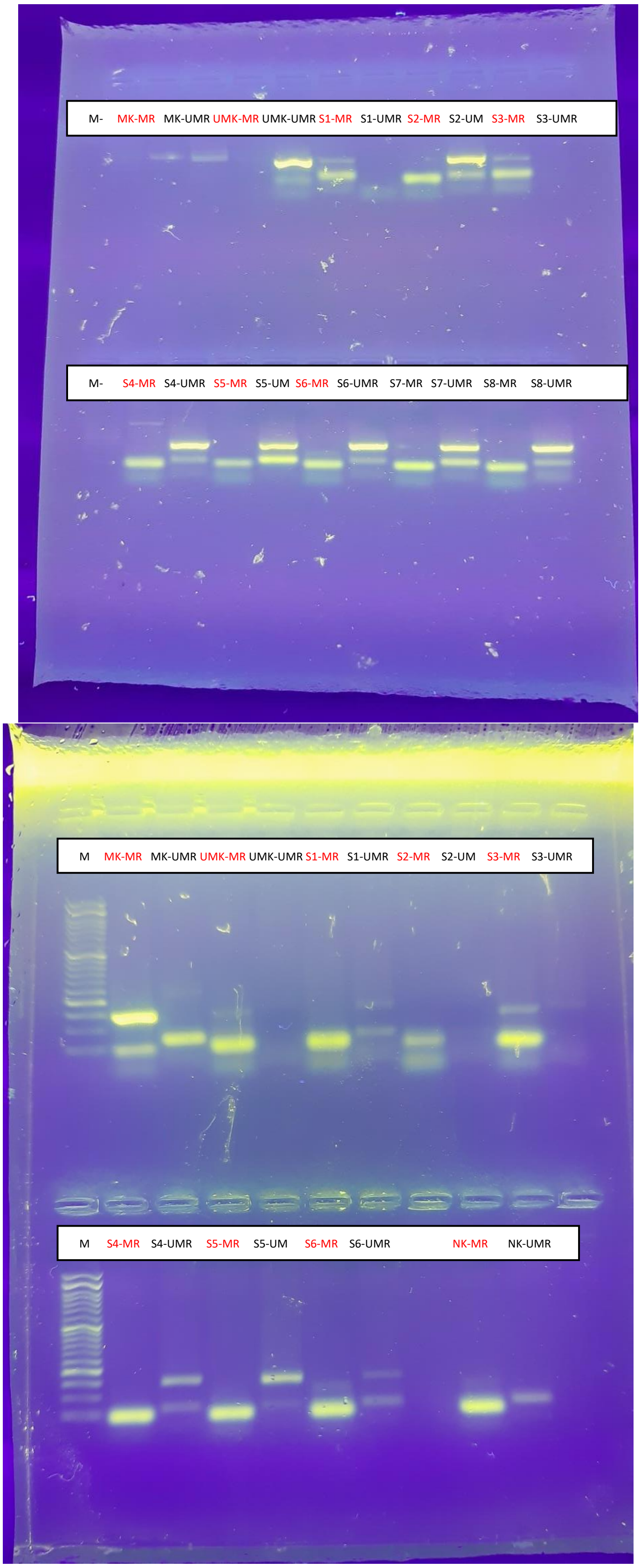

Figure S1. M = 50 bp molecular marker; MK-MR = methylated control methylated reaction; MK-UMR = methylated control unmethylated reaction; UMK-MR = unmethylated control methylated reaction; UMK-UMR = unmethylated control unmethylated reaction; S(1–6)-MR = sample (1–6) methylated reaction; S(1–6)-UMR = sample (1–6) unmethylated reaction; NK-MR = negative control methylated reaction; NK-UMR = negative control unmethylated reaction

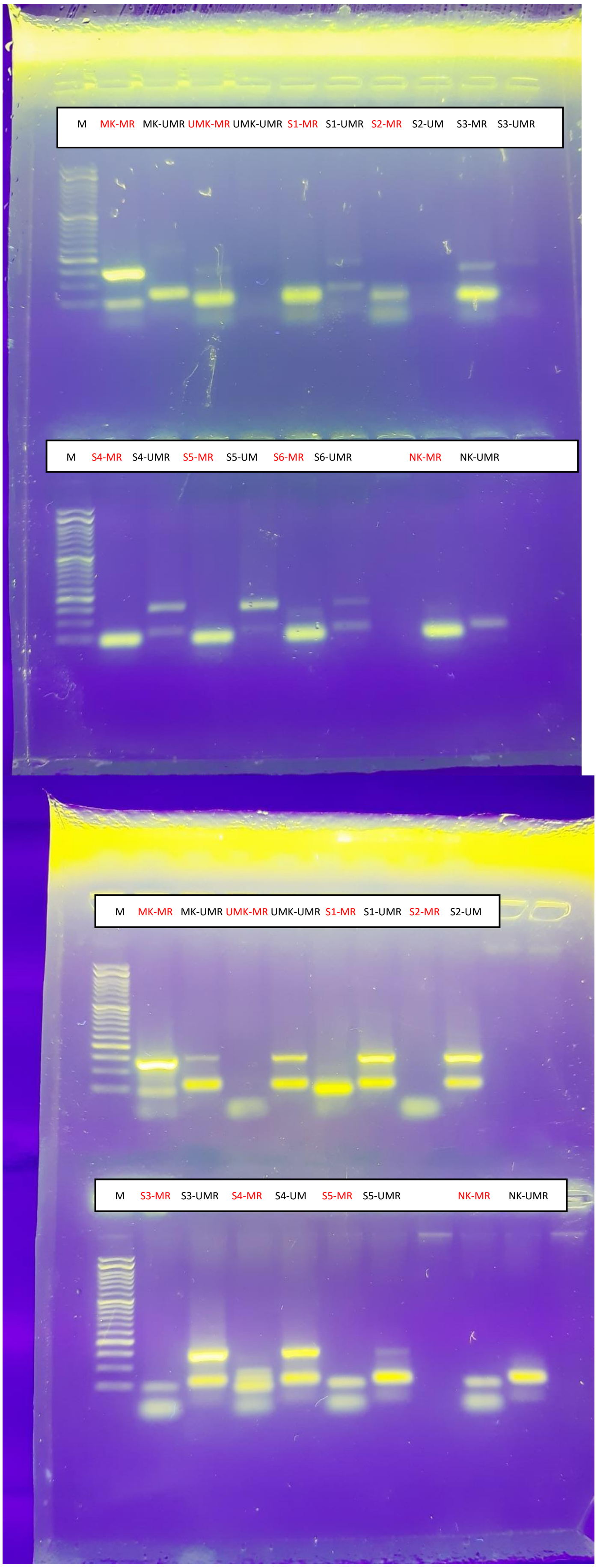

Figure S2. M = 50 bp molecular marker; MK-MR = methylated control methylated reaction; MK-UMR = methylated control unmethylated reaction; UMK-MR = unmethylated control methylated reaction; UMK-UMR = unmethylated control unmethylated reaction; S(1–6)-MR = sample (1–6) methylated reaction; S(1–6)-UMR = sample (1–6) unmethylated reaction; NK-MR = negative control methylated reaction; NK-UMR = negative control unmethylated reaction

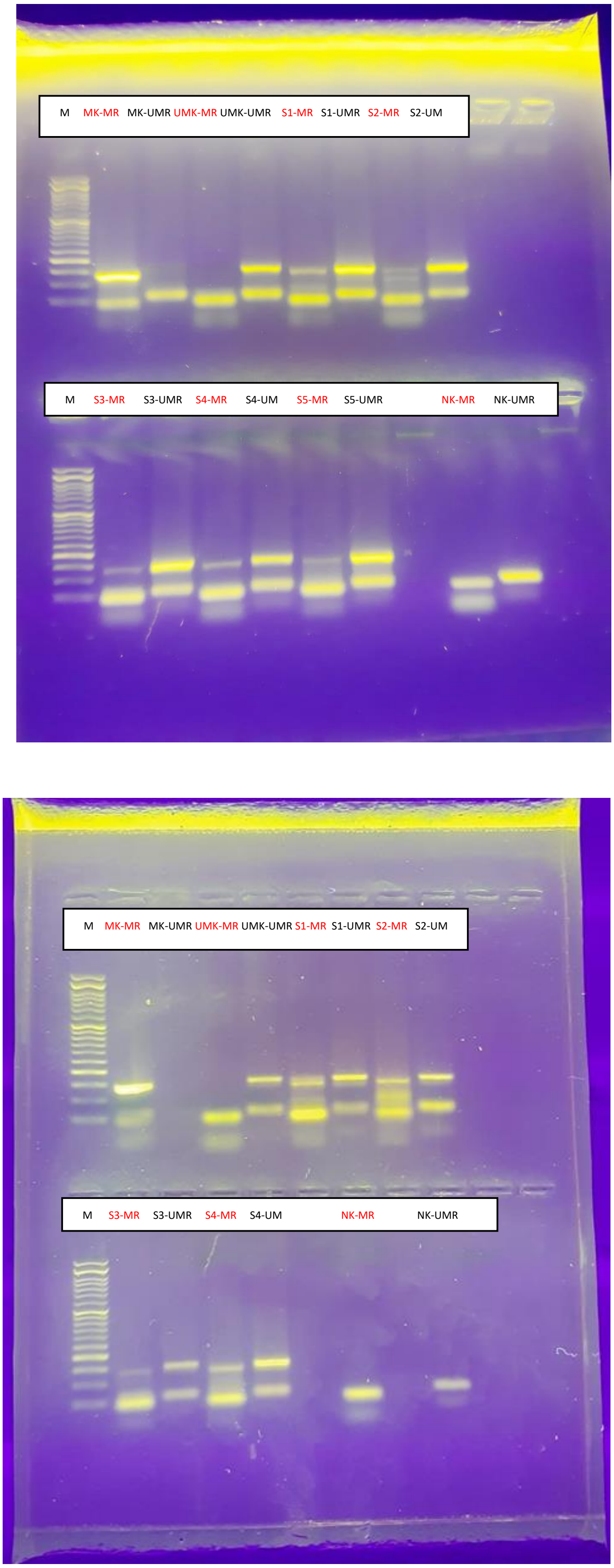

Figure S3. M = 50 bp molecular marker; MK-MR = methylated control methylated reaction; MK-UMR = methylated control unmethylated reaction; UMK-MR = unmethylated control methylated reaction; UMK-UMR = unmethylated control unmethylated reaction; S(1–6)-MR = sample (1–6) methylated reaction; S(1–6)-UMR = sample (1–6) unmethylated reaction; NK-MR = negative control methylated reaction; NK-UMR = negative control unmethylated reaction

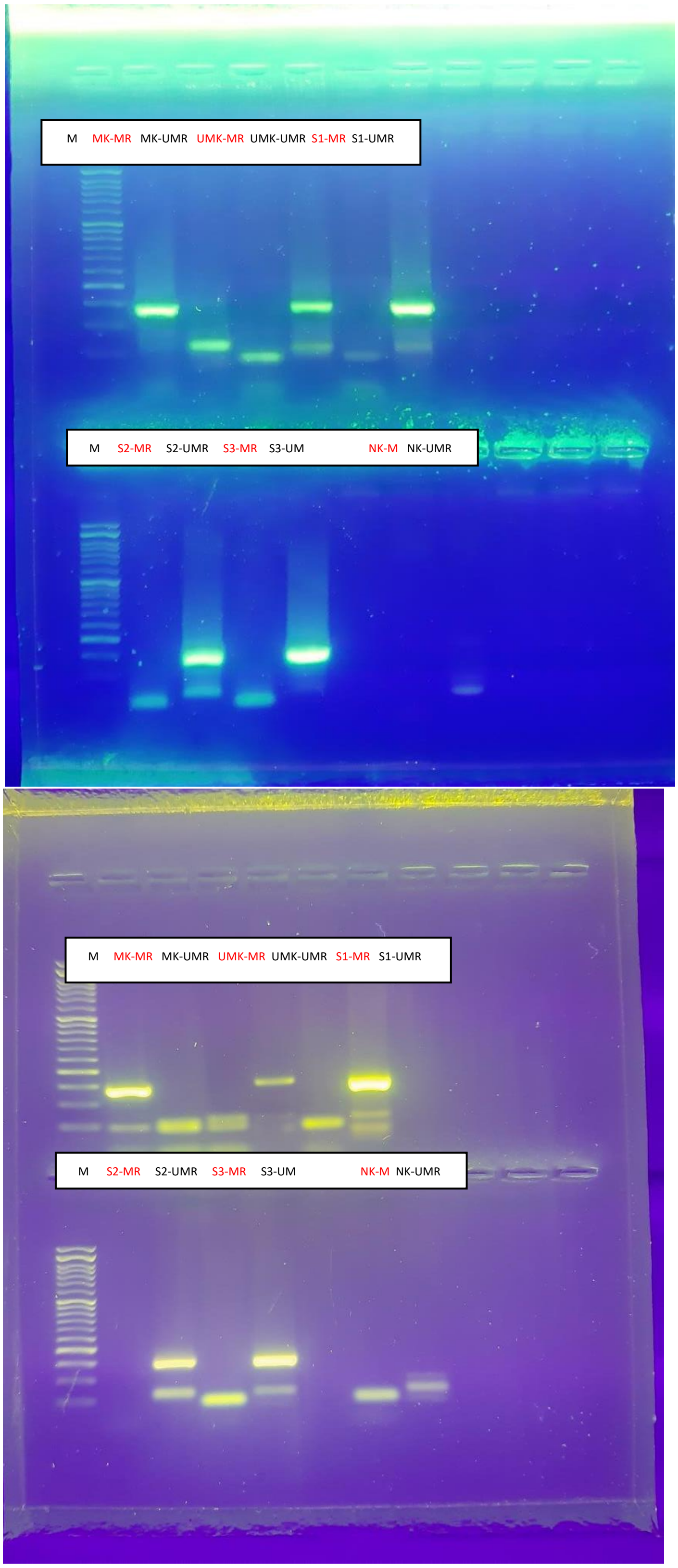

Figure S4. M = 50 bp molecular marker; MK-MR = methylated control methylated reaction; MK-UMR = methylated control unmethylated reaction; UMK-MR = unmethylated control methylated reaction; UMK-UMR = unmethylated control unmethylated reaction; S(1–6)-MR = sample (1–6) methylated reaction; S(1–6)-UMR = sample (1–6) unmethylated reaction; NK-MR = negative control methylated reaction; NK-UMR = negative control unmethylated reaction
